# Potential Anti-Amnesic Activity of a Novel Multimodal Derivative of Salicylamide, JJGW08, in Mice

**DOI:** 10.3390/ph16030399

**Published:** 2023-03-06

**Authors:** Elżbieta Żmudzka, Klaudia Lustyk, Kinga Sałaciak, Agata Siwek, Jolanta Jaśkowska, Marcin Kołaczkowski, Jacek Sapa, Karolina Pytka

**Affiliations:** 1 Department of Social Pharmacy, Faculty of Pharmacy, Jagiellonian University Medical College, 30-688 Krakow, Poland; 2 Department of Pharmacodynamics, Faculty of Pharmacy, Jagiellonian University Medical College, 30-688 Krakow, Poland; 3 Department of Pharmacobiology, Faculty of Pharmacy, Jagiellonian University Medical College, 30-688 Krakow, Poland; 4 Department of Organic Chemistry and Technology, Faculty of Chemical and Engineering and Technology, Cracow University of Technology, 31-155 Krakow, Poland; 5 Department of Medicinal Chemistry, Faculty of Pharmacy, Jagiellonian University Medical College, 30-688 Krakow, Poland

**Keywords:** anti-amnesic effect, long-term memory, cognition, antidepressant-like activity, serotonin receptors

## Abstract

Memory impairments constitute a significant problem worldwide, and the COVID-19 pandemic dramatically increased the prevalence of cognitive deficits. Patients with cognitive deficits, specifically memory disturbances, have underlying comorbid conditions such as schizophrenia, anxiety, or depression. Moreover, the available treatment options have unsatisfactory effectiveness. Therefore, there is a need to search for novel procognitive and anti-amnesic drugs with additional pharmacological activity. One of the important therapeutic targets involved in the modulation of learning and memory processes are serotonin receptors, including 5-HT_1A_, 5-HT_6_, and 5-HT_7_, which also play a role in the pathophysiology of depression. Therefore, this study aimed to assess the anti-amnesic and antidepressant-like potential of JJGW08, a novel arylpiperazine alkyl derivative of salicylamide with strong antagonistic properties at 5-HT_1A_ and D_2_ receptors and weak at 5-HT_2A_ and 5-HT_7_ receptors in rodents. First, we investigated the compound’s affinity for 5-HT_6_ receptors using the radioligand assays. Next, we assessed the influence of the compound on long-term emotional and recognition memory. Further, we evaluated whether the compound could protect against MK-801-induced cognitive impairments. Finally, we determined the potential antidepressant-like activity of the tested compound. We found that JJGW08 possessed no affinity for 5-HT_6_ receptors. Furthermore, JJGW08 protected mice against MK-801-induced recognition and emotional memory deficits but showed no antidepressant-like effects in rodents. Therefore, our preliminary study may suggest that blocking serotonin receptors, especially 5-HT_1A_ and 5-HT_7_, might be beneficial in treating cognitive impairments, but it requires further investigation.

## 1. Introduction

Memory is fundamental to everyday life, and cognitive impairments significantly deteriorate normal functioning. The World Health Organization reports that dementia, a loss of cognitive functioning, affects more than 55 million people worldwide and constitutes the seventh leading cause of death [1]. The prevalence of memory deficits increased due to the COVID-19 pandemic, which elevated the number of reported problems with cognition, including brain fog, forgetfulness, and difficulty concentrating [2,3,4,5]. Furthermore, memory impairments can constitute a separate disorder, such as dementia or Alzheimer’s disease, but they can also accompany other conditions, such as depression or schizophrenia, worsening their course and increasing the risk of developing resistance to the treatment [6,7,8,9,10,11,12,13]. Moreover, not all types of memory are equally impaired in the above-mentioned conditions [6,14,15]; therefore, it is important to investigate the effects of novel compounds on various types of memory. Unfortunately, the effectiveness of available treatment options, mainly limited to acetylcholinesterase inhibitors or NMDA receptor antagonists, is disappointing and insufficient [16,17]. Therefore, there is a need to look for novel procognitive and anti-amnesic drugs with additional antidepressant-like activity.

Among various drug targets, especially 5-HT_1A_, 5-HT_6_, and 5-HT_7_ receptors are important for learning and memory as they are localized in brain areas involved in cognitive processes (such as the hippocampus, prefrontal cortex, or amygdala [18,19,20]). Interestingly, their role in memory depends on each other’s activity. Stimulating 5-HT_7_ receptors with the subsequently reduced 5-HT_1A_ receptor-mediated transmission facilitates emotional memory in mice [21]. Furthermore, various research suggests that agonists and antagonists of 5-HT_1A_ [22,23,24], 5-HT_6_ [25,26,27], and 5-HT_7_ receptors [28,29,30,31,32] are effective in the treatment of learning and memory disorders. Both agonists and antagonists of the above receptors showed not only procognitive [22,33,34] but also anti-amnesic properties, reversing memory deficits of the glutamatergic or cholinergic origin [23,25,28,30,35]. However, these contradictory research results may be associated with different types of memory assessed, as well as the stage of memory formation. Nevertheless, 5-HT_1A_, 5-HT_6_, and 5-HT_7_ receptors and their ligands are worth studying in memory disorders with different etiology. Moreover, ligands of these receptors also play a significant role in depression treatment. Many studies confirmed the potential antidepressant-like activity of antagonists of 5-HT_1A_ [36,37], 5-HT_6_ [38,39,40], and 5-HT_7_ [41,42,43,44,45] receptors. Therefore, it is also reasonable to investigate their antidepressant-like properties.

Studies indicated that compounds with 2-methoxyphenylpiperazine fragments bind to various serotonin receptors, particularly 5-HT_1A_ and 5-HT_7_ [37,46,47]. Moreover, such compounds showed a promising pharmacological profile in animals, such as anti-amnesic and procognitive effects [48,49], as well as antidepressant-like properties [47,50,51]. Additionally, a study by Jaśkowska et al. demonstrated that salicylamide derivatives with arylpiperazine moiety could bind strongly with serotonin receptors, especially 5-HT_1A_ and 5-HT_7_ receptors [52]. Therefore, we hypothesized that salicylamide derivative with 2-methoxyphenyl moiety could improve rodents’ cognition and may alleviate depressive-like behavior.

Given the optimistic assumptions, this study aimed to assess the potential anti-amnesic and anti-depressant-like effects of JJGW08, a novel arylpiperazine alkyl derivative of salicylamide, which in our earlier studies showed strong antagonistic properties at 5-HT_1A_ and D_2_ receptors and weak at 5-HT_2A_ and 5-HT_7_ receptors as well as the potential antipsychotic- and anxiolytic-like activity in rodents [53]. We assessed whether the compound, when given alone, affected the emotional and recognition memory using the step-through passive avoidance and object recognition tests, respectively. Moreover, we evaluated whether the compound reversed cognitive impairments caused by MK-801, an antagonist of NMDA receptors. Finally, we investigated its potential antidepressant-like activity in the forced swim and tail suspension tests in rodents. 

## 2. Results

### 2.1. JJGW08 Showed No Affinity for 5-HT_6_ Receptors

The studied compound possessed no affinity for 5-HT_6_ receptors and did not bind to the receptor at the concentration 10^−5^M, whereas the *p*Ki value for methiotepin, a reference compound, was 8.48 ± 0.04.

### 2.2. JJGW08 Did Not Disturb Long-Term Memory in Naïve Mice in the Step-Through Passive Avoidance Task

**Figure 1:**
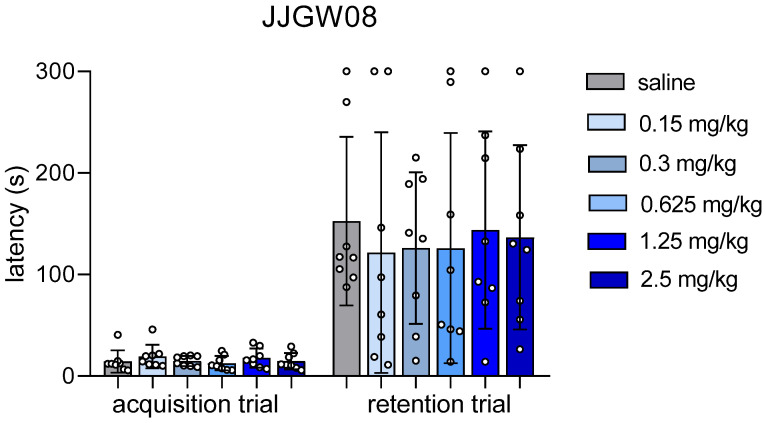
The influence of JJGW08 on the latency in the step-through passive avoidance task in mice. The experiment consisted of two sessions, i.e., the acquisition and the retention trial. In the acquisition trial mice were placed individually in the light chamber of the apparatus, with the door opening after 30 s. When the animal crossed to the dark chamber, the door closed, and the animal was punished with an electric shock (0.8 mA, 2 s). JJGW08 was administered intraperitoneally (*ip*) 30 min before the start of the experiment. The control group received *ip* 0.9% NaCl solution. On the second day of the test, mice were placed again in the light chamber, and the latency was measured for a maximum of 300 s (without an electrical impulse). Values are expressed as means ± SD, n = 8 mice per group. Statistical analysis: two-way ANOVA with repeated measures (Bonferroni *post hoc*).

JJGW08 did not influence the latency in the acquisition trial, but it significantly increased the latency in the retention trial at all tested doses compared to the acquisition session. Statistical analysis showed significant time effect (F(1,42) = 74.30, *p* < 0.0001), but no influence of the compound (F(5,42) = 0.115, *p* = 0.988) and no interaction (F(5,42) = 0.141, *p* = 0.982) (Figure 1).

### 2.3. JJGW08 Reversed Cognitive Disturbances after MK-801 Administration in Mice in the Step-Through Passive Avoidance Task

**Figure 2:**
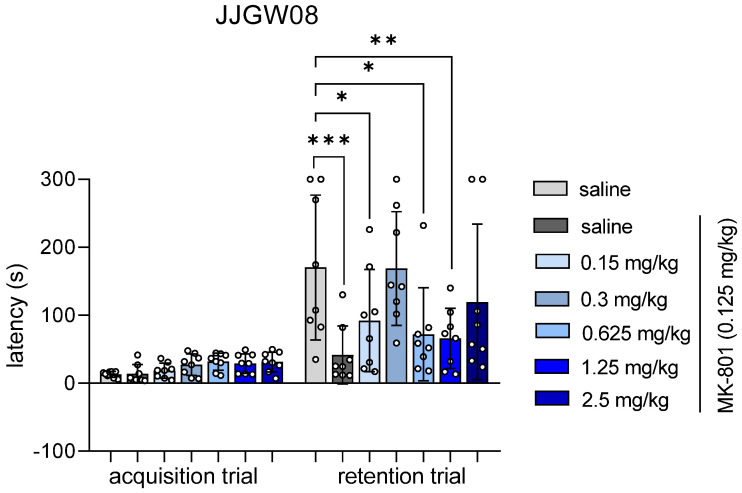
The influence of JJGW08 on the latency after the MK-801 administration in the step-through passive avoidance task in mice. The experiment consisted of two sessions, i.e., the acquisition and retention trial. In the acquisition trial, mice were placed individually in the light chamber of the apparatus, with the door opening after 30 s. When the animal crossed to the dark chamber, the door closed, and the animal was punished with an electric shock (0.8 mA, 2 s). JJGW08 was administered intraperitoneally (*ip*) 30 min before the test, while MK-801 (0.125 mg/kg) was administered *ip* 15 min before the start of the experiment to induce memory impairments. The control group received *ip* 0.9% NaCl solution in two injections or 0.9% NaCl solution and MK-801 (0.125 mg/kg; *ip*). On the second day of the test, mice were placed again in the light chamber, and the latency was measured for a maximum of 300 s (without electrical impulse). Values are expressed as means ± SD, n = 8 mice per group. Statistical analysis: two-way ANOVA with repeated measures (Bonferroni *post hoc*) * *p* < 0.05, ** *p* < 0.01, *** *p* < 0.001.

The studied compound, MK-801, did not influence the latency in the acquisition trial. JJGW08 reversed MK-801-induced memory impairments at the doses of 0.3 mg/kg and 2.5 mg/kg by increasing the latency in the retention session. Statistical analysis showed significant time effect (F(1,48) = 53.87, *p* < 0.0001), significant effect of the compound (F(6,49) = 3.435, *p* = 0.007) and interaction (F(6,48) = 3.514, *p* < 0.006) (Figure 2).

### 2.4. JJGW08 Did Not Disturb Long-Term Memory in Naïve Mice in the Object Recognition Test

**Figure 3:**
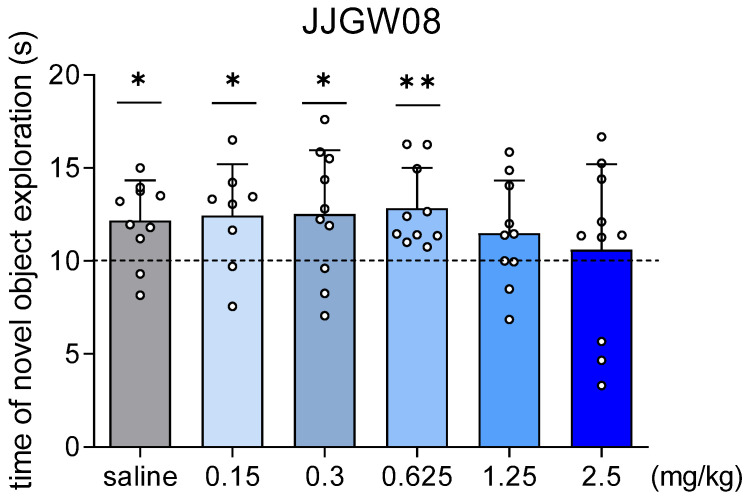
The influence of JJGW08 on the novel object exploration time in mice. The experiment consisted of two sessions. On the first day, mice were placed and left in cages until they reached a total exploration time of 20 s for both identical objects, but no longer than 10 min. JJGW08 was administered *ip* 30 min before the start of the experiment. The control group received *ip* 0.9% NaCl solution. On the second day, mice were placed again in cages, where one object was changed to a new one. The mice remained in the cage until they reached a total exploration time of 20 s for both objects, but no longer than 10 min. Values are expressed as means ± SD, n = 8–10 mice per group. Statistical analysis: one-sample *t*-test * *p* < 0.05, ** *p* < 0.01 vs. chance level = 10 s.

JJGW08 at the doses 0.15, 0.3, and 0.625 mg/kg increased the exploration of the novel object, and the exploration time was significantly higher than the chance level of 10 s (Figure 3).

### 2.5. JJGW08 Reversed Cognitive Disturbances after the MK-801 Administration in Mice in the Object Recognition Test

**Figure 4:**
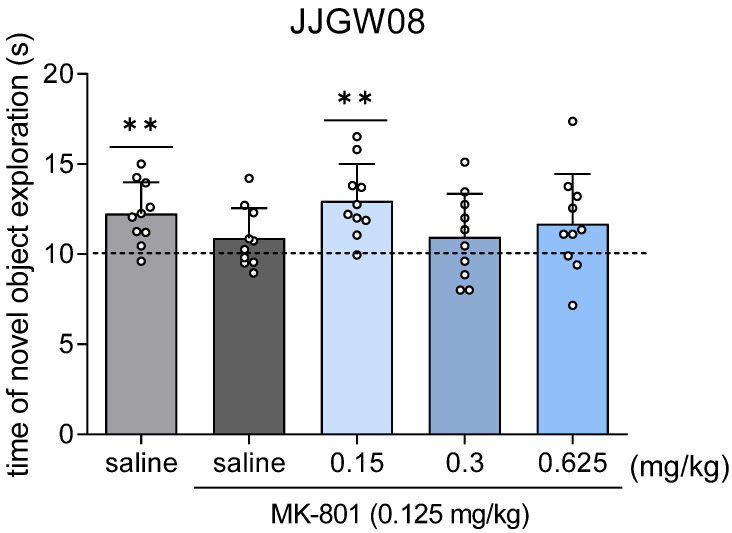
The influence of JJGW08 on the novel object exploration time after the MK-801 administration in mice. The experiment consisted of two sessions. On the first day, mice were placed and left in cages until they reached a total exploration time of 20 s for both identical objects, but no longer than 10 min. The tested compound was administered intraperitoneally (*ip)* 30 min before, while MK-801 (0.125 mg/kg) was administered *ip* 15 min before the start of the experiment to induce memory impairments. The control group received 0.9% NaCl solution *ip* in two injections or 0.9% NaCl solution *ip* and MK-801 (0.125 mg/kg; *ip*). On the second day, mice were placed again in cages, where one object was changed to a new one. The mice remained in the cage until they reached a total exploration time of 20 s for both objects, but no longer than 10 min. Values are expressed as means ± SD, n = 8–10 mice per group. Statistical analysis: one-sample *t*-test ** *p* < 0.01 vs. chance level = 10.

In mice administered with MK-801 alone, the novel object exploration time did not differ significantly from the chance level. JJGW08 at the dose of 0.15 mg/kg significantly increased the exploration of the novel object compared to the chance level of 10 s (Figure 4). 

### 2.6. JJGW08 Did Not Decrease the Immobility Time in the Forced Swim and Tail Suspension Tests in Mice

**Figure 5:**
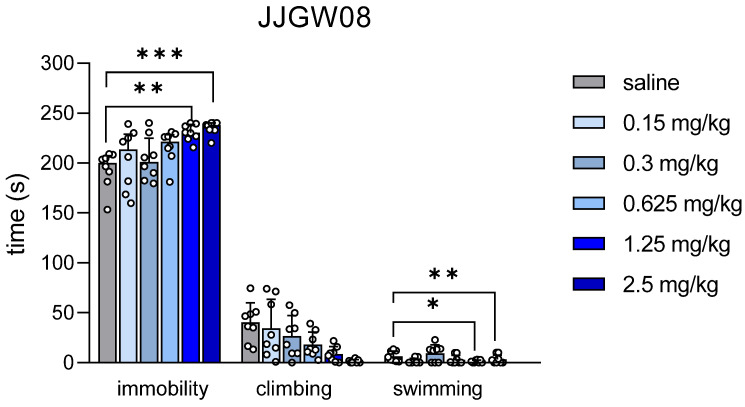
The effect of JJGW08 on the immobility, climbing, and swimming time in the forced swim test in mice. Mice were placed in water tanks, and the immobility time, as well as swimming and climbing time, were measured for 4 min (after a 2-min adaptation period). JJGW08 was administered intraperitoneally (*ip*) 30 min before the test. The control group received an injection of 0.9% NaCl (*ip*). Values are expressed as means ± SD in case of one-way ANOVA (climbing and swimming) or medians with interquartile range in case of Kruskal–Wallis test (immobility), n = 8–10 mice per group. Statistical analysis: one-way ANOVA (Newman–Keuls *post hoc*) or Kruskal–Wallis (Dunn’s *post hoc*), * *p* < 0.05, ** *p* < 0.01, *** *p* < 0.001 vs. control group.

JJGW08 increased the immobility time at the doses 1.25 mg/kg and 2.5 mg/kg (H(6,48) = 22.56, *p* = 0.0004) and decreased the swimming time (F(6,48) = 5.705, *p* = 0.0015) without affecting climbing time (F(5,42) = 3.174, *p* = 0.0161) in the forced swim test in mice (Figure 5).

**Figure 6:**
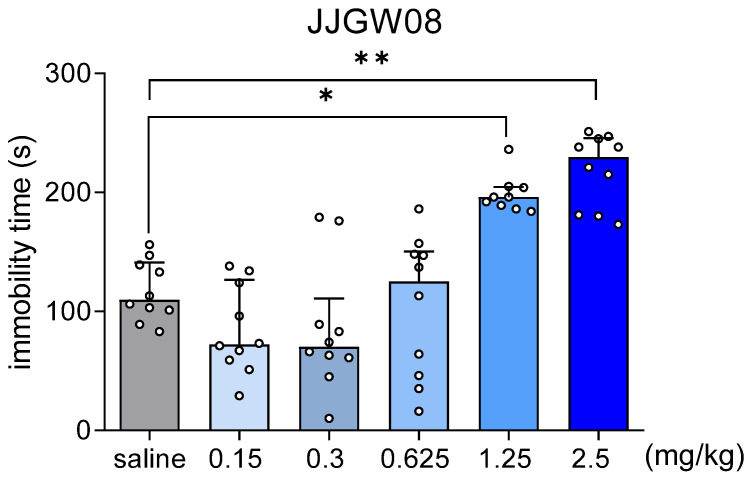
The effect of JJGW08 on the immobility time in the tail suspension test in mice. Mice were suspended by the tail (50 cm above the ground) with adhesive tape (1 cm wide), and the immobility time was measured for 6 min. JJGW08 was administered intraperitoneally (*ip*) 30 min before the test. The control group received an injection of 0.9% NaCl (*ip*). Values are expressed as medians with interquartile range, n = 10 mice per group. Statistical analysis: Kruskal–Wallis (Dunn’s *post hoc*), * *p* < 0.05, ** *p* < 0.01 vs. control group.

Moreover, JJGW08 increased the immobility time in the tail suspension test at the doses 1.25, and 2.5 mg/kg by 70 and 87%, respectively (H(6,59) = 39.02, *p* < 0.0001) (Figure 6).

### 2.7. JJGW08 Decreased the Locomotor Activity in Mice

JJGW08 decreased the locomotor activity in mice at the dose 2.5 mg/kg in the 6-min session (F(5,44) = 5.298, *p* = 0.0007), and 4-min session (F(5,44) = 5.654, *p* = 0.0004) (Table 1).

## 3. Discussion

In this study, we found that JJGW08, which is a novel arylpiperazine alkyl derivative of salicylamide, with strong antagonistic properties at the 5-HT_1A_ and D_2_ receptors and weak at the 5-HT_2A_ and 5-HT_7_ receptors, as well as the antipsychotic- and anxiolytic-like activity, possessed no affinity towards 5-HT_6_ receptors. Furthermore, behavioral studies demonstrated that JJGW08 did not impair long-term emotional or recognition memory acquisition in mice. Notably, the compound protected mice against MK-801-induced recognition and emotional memory deficits. Finally, the tested compound did not show antidepressant-like effects in mice.

Serotonergic neurons are widely distributed in the brain with high density in the hippocampus and prefrontal cortex, areas strongly associated with cognition [54,55,56] and depression [57]. Thus, it is unsurprising that serotonin and its receptors play a significant role in learning and memory processes, as well as mood regulation. Interestingly, among serotonin receptors, the 5-HT_1A_, 5-HT_6_, and 5-HT_7_ subtypes are of particular interest as targets for the treatment of memory deficits (reviewed in [58] and [59]) and depressive states [60]. Our previous studies revealed that JJGW08 was a potent antagonist of 5-HT_1A_ and D_2_ receptors and a weak antagonist of 5-HT_2A_ and 5-HT_7_ receptors [53]. Considering the pivotal role of the serotonin 5-HT_6_ receptor modulation in cognitive processes [61,62,63,64,65], we first investigated the compound’s affinity for the 5-HT_6_ receptors using radioligand binding studies. We showed that JJGW08 did not demonstrate any affinity for the 5-HT_6_ receptors. Nevertheless, since JJGW08 targets other serotonin receptors, it encouraged us for further investigation. Studies indicated that the blockade of 5-HT_1A_ and 5-HT_7_ receptors resulted in anti-amnesic effects in rodents [32,48,66,67] as well as antidepressant-like activity in animals (reviewed in [60]). Thus, the blockade of both these receptors might be beneficial in restoring normal cognitive processes and alleviating depressive-like symptoms in rodents.

Bearing that in mind, we examined the effects of JJGW08 on the acquisition phase of long-term emotional memory in the step-through passive avoidance test in mice. This test involves analysis of conflict behavior, such as avoidance of aversive stimuli, i.e., electric shock. The animal must inhibit its natural preference for the dark compartment, where it receives an electric shock during the familiarization phase and stays in the bright chamber [68]. The results indicate that JJGW08 significantly increased the latency to enter the dark compartment, which suggests that it did not negatively influence long-term emotional memory acquisition. Next, we used the same behavioral assay to investigate whether JJGW08 would protect against MK-801-induced emotional memory impairment. We used MK-801, an NMDA antagonist, to impair memory, as the NMDA receptors are involved with cognitive processes, especially the long-term potentiation [69]. Our results demonstrated that the administration of MK-801 in the step-through passive avoidance test caused a significant decrease in the latency to enter the dark compartment. However, the pretreatment with JJGW08 showed a potential anti-amnesic effect by increasing the time to enter the dark chamber. Interestingly, we observed a U-shaped dose effect (the highest dose of 2.5 mg/kg and the dose of 0.3 mg/kg were effective). Such an effect is a common phenomenon in studies on cognition and is probably multifactorial. In the case of JJGW08, it might be due to the interaction with other, not yet tested biological targets, which might influence the overall effect of the compound on emotional memory.

Knowing the positive influence on emotional memory impairments, we assessed the effect of JJGW08 on recognition memory using the object recognition test in mice. This behavioral test is based on rodents’ tendency to explore a novel object longer than a familiar one [70]. The results showed that JJGW08 impaired memory at higher doses, whereas lower doses did not affect recognition memory acquisition. Thus, for studies with MK-801, we chose lower doses of the compound. An antagonist of NMDA receptors induced recognition memory deficits, decreasing the exploration time of the new object. Interestingly, JJGW08 protected from recognition memory deficits only at the lowest dose tested (i.e., 0.15 mg/kg). As mentioned earlier, this effect is a common phenomenon in neuropharmacological research, especially for multimodal compounds, probably due to varying receptor occupancy at various doses or dose-dependent optimal receptor saturation. Our study suggests that at lower doses, JJGW08 shows potential anti-amnesic properties, but further studies are necessary to confirm our speculations.

Since the serotonin receptors also play a significant role in depression, in the final step of our study, we evaluated the antidepressant-like effects of JJGW08 using the forced swim and tail suspension tests in mice, which are the most commonly used tests to evaluate antidepressant-like effects of new compounds [71]. JJGW08 demonstrated no antidepressant-like properties in both tests, and conversely, at two doses (1.25 and 2.5 mg/kg), it increased the immobility time of mice. These results might be due to the sedative properties of JJGW08, as at the highest dose tested (2.5 mg/kg), the compound reduced the locomotor activity of animals. Sedation is a common side effect of various drugs acting in the central nervous system, for example, blocking H_1_ or α_1_ receptors or activating µ-opioid receptors [72,73,74,75]. Therefore, we might suspect that JJGW08 at the highest tested doses may bind to other types of receptors responsible for this undesirable effect. Nevertheless, this issue requires further studies to explain the observed effects.

Our results align with the findings of other research groups, in which either the 5-HT_1A_ and/or 5-HT_7_ antagonists show anti-amnesic properties (reviewed in [76] and [77]). Much evidence indicates the interaction between serotoninergic and glutamatergic neurotransmission in learning and memory processes [78]. Studies showed the improvement of memory deficits due to the blockade of NMDA receptors or glutamatergic lesions after the treatment with serotonin 5-HT_7_ antagonists [79,80] as well as 5-HT_1A_ [81,82,83] and 5-HT_6_ [25] ago- or antagonists in behavioral tests in animals. For example, Bonaventure et al. demonstrated that SB-269970, a selective 5-HT_7_ antagonist, reversed memory deficits due to NMDA receptor hypofunction by the selective normalization of glutamatergic neurotransmission [84]. Similarly, Harder and Ridley showed that WAY 100 635 (a 5-HT_1A_ receptor antagonist) alleviated cognitive impairment induced by MK-801 in monkeys [85]. Since JJGW08 is an antagonist of 5-HT_1A_ and 5-HT_7_ receptors, we can speculate that the observed protection against MK-801-induced memory deficits may be due to the blockade of these receptors. Nevertheless, further studies are required to confirm this assumption.

Altogether, our study suggests that dual 5-HT_1A_ and 5-HT_7_ antagonists might help treat memory deficits. However, we need to emphasize that JJGW08 antagonized the 5-HT_1A_ more strongly than the 5-HT_7_ receptor, which suggests that the 5-HT_1A_ receptor may play a leading role in the potential anti-amnesic effect. Nevertheless, further studies are necessary to determine the full potential of this compound group.

Our study has some limitations. First, we should evaluate the effect of JJGW08 on learning and memory after repeated administration. Moreover, we should also investigate the influence of JJGW08 on other types of memory. Finally, it is also necessary to determine the pharmacokinetic profile of JJGW08 and verify its ability to cross the blood–brain barrier.

## 4. Materials and Methods

### 4.1. Drugs

The studied compound 2-{5-[4-(2-methoxyphenyl)piperazin-1-yl]pentoxy}benzamide hydrochloride (JJGW08) was synthesized in the Department of Organic Chemistry and Technology, Faculty of Chemical and Engineering and Technology, Cracow University of Technology. The synthesis and biological properties of the compound were described earlier [52]. 

JJGW08 was dissolved in saline (0.9 % NaCl, Polpharma, Starogard Gdańsk, Poland) and administered intraperitoneally (*ip)* 30 min before each behavioral test. The chemicals used in radioligand studies, i.e., methiothepin (Sigma-Aldrich, Darmstadt, Germany), were dissolved in saline. MK-801 (Sigma-Aldrich, Darmstadt, Germany) was dissolved in saline and administered *ip* 15 min before experiments. The control groups received 0.9% NaCl solution *ip* 30 min prior to testing. The studied compound was tested in in vivo experiments at the dose range of 0.625–2.5 mg/kg, which was selected based on our previous experiments [53]. If the effect of the lowest dose, i.e., 0.625 mg/kg, was still statistically significant, we reduced the dose by half until the observed activity disappeared.

### 4.2. Animals

All experiments were performed on adult male CD-1 mice weighing 18–21 g, obtained from an accredited house at the Faculty of Pharmacy, Jagiellonian University Medical College, Krakow, Poland. Mice were kept in groups of 10 in plastic cages (37 cm × 21 cm × 15 cm) in a controlled environment (i.e., constant room temperature (22 ± 2 °C), adequate humidity (40–60%), 12 h light/dark cycle) with *ad libitum* access to standard pellet food and filtered tap water. 

All experimental procedures were performed between 8 a.m. and 4 p.m. Animals were selected randomly for treatment groups and each group consisted of 8–10 mice. All injections were administered in a volume of 10 mL/kg. The animals were used only once in each test, and immediately after each experiment, they were euthanized.

Procedures involving animals were conducted according to current European Community and Polish legislation on animal experimentation and were approved by the Local Ethical Committee for Animal Experiments at the Jagiellonian University in Krakow (approval codes: 102/2016 and 170/2018).

### 4.3. Radioligand Binding Assays

Radioligand binding assays were performed using membranes from CHO-K1 cells, which were stably transfected with the human 5-HT_6_ receptor. The assay procedures were conducted according to the slightly modified method described by Sałaciak and colleagues [37].

Binding experiments were performed in 96-well microplates, and the reaction mix included a solution of the tested compound, radioligand, and diluted membranes or the tissue suspension. Specific assay conditions for each target are shown in Table 2. The reaction was discontinued by the rapid filtration through GF/B or GF/C filter mate using an automated harvester system FilterMate Harvester (PerkinElmer, Boston, MA, USA). The filter mates were dried at 37 °C in a forced air fan incubator, and then solid scintillator MeltiLex was melted on filter mates at 90 °C for 5 min. Radioactivity was counted in the MicroBeta2 scintillation counter (PerkinElmer, Boston, MA, USA) at approximately 30% efficiency. The concentrations of the analyzed compound ranged from 10^–10^ to 10^–5^ M. The inhibitory constant (Ki) was estimated using GraphPad Prism 5.0 (GraphPad Software, San Diego, CA, USA). A single assay was performed with each compound concentration in duplicate, and the whole assay was repeated in three independent experiments. Inhibition constants (Ki) were calculated according to the equation of Cheng and Prusoff [86].

### 4.4. Step-Through Passive Avoidance Task

The step-through passive avoidance task was performed according to the method previously described [36,68]. The apparatus for the step-through passive avoidance task consisted of two compartments (i.e., bright, and dark), separated by an automated sliding door (LE872, Bioseb, Vitrolles, France). For the acquisition session, mice were placed individually in a bright compartment (20 cm × 21 cm × 20 cm, 1000 lx) with a closed door to a smaller, dark compartment (7.3 cm × 7.5 cm × 14 cm, 10 lx) equipped with an electric grid floor. A total of 30 s after placing the animal in the bright compartment, the door to the dark compartment was opening. If the mouse entered the dark compartment, the door closed immediately, and the rodent was punished by an electric foot shock (0.8 mA for 2 s). The mice that did not enter the dark compartment within the next 50 s were excluded from the study. On the following day (24 h later), animals were placed again in the bright compartment for 300 s (retention session) with the difference that after entering the dark compartment mice were not receiving the electric shock. The latency to enter the dark compartment was measured. The tested compound was administered *ip* 30 min before the acquisition trial. To induce memory impairments, MK-801 (0.125 mg/kg) was administered *ip* 15 min before the experiment. Control groups were injected *ip* with saline or with saline and MK-801. 

### 4.5. Object Recognition Test

The test was performed according to the method described earlier [87,88] and consisted of a familiarization and test session. In the familiarization session, mice were placed individually in the cage (35 cm × 35 cm × 35 cm) and remained there until the total exploration time of both identical objects (2 towers of Lego bricks or 2 bottles filled with sand) was 20 s or 10 min has passed. Animals that did not meet the criteria were eliminated from further study. After 24 h, mice were placed again in the cage, with the difference that one object was replaced with the new one. As before, mice remained in the cage until the total exploration time of 20 s, but not longer than 10 min. We recorded the time of exploration of both new and old objects. However, for further analysis, we used only the exploration time of the new object. The tested compound was administered *ip* 30 min before the familiarization phase to assess its influence on recognition memory. To induce memory disturbances, MK-801 (0.125 mg/kg) was administered *ip* 15 min before the experiment. Control groups were injected *ip* with saline or with saline and MK-801.

### 4.6. Forced Swim Test

The experiment was performed on mice according to the method described by Porsolt et al. and as previously described [36,89]. Mice were placed individually in glass cylinders (height 25 cm, diameter 10 cm) filled with water at 24 ± 1 °C to a depth of 10 cm and left there for 6 min. Following a 2 min habituation period, the total time spent immobile was recorded during the next 4 min. The animal was regarded as immobile when it remained floating passively in the water, making only small movements to keep its head above the water. The experiments were video-recorded and scored using elevenmaze.com software (Eleven Products Sp. z o.o., Krakow, Poland) by a trained observer blind to the treatment. 

### 4.7. Tail Suspension Test

The experiment was carried out on mice according to the method described by Steru et al. and as previously described [90,91]. The mice were suspended by their tails using a medical adhesive tape at a height of 50 cm above a flat surface, in such a position that they cannot escape or hold on to the nearby surfaces. The total time of immobility was measured during the 6-min test period. Immobility was defined as the animal hanging passively without limb movement. The experiments were performed by a trained observer blind to the treatment.

### 4.8. Spontaneous Locomotor Activity in Mice

The locomotor activity of mice was measured as previously described [47], using actometers, i.e., plastic Opto M3 cages (22 cm × 12 cm × 13 cm) connected to a computer with MultiDevice Software v.1.30 (Columbus Instruments, Columbus, OH, USA). The experimental cages were equipped with infrared sources on one side and sensors receiving the emitted rays on the other side of the cage. The crossing of each beam of infrared rays was classified as motor activity. Each mouse was placed individually in a cage for 30 min habituation period (directly after administration of the studied compound), and then the number of photobeam crossings was recorded (ambulation). Locomotor activity was evaluated every 1 min for 6 min (6 min observation for the tail suspension test, and 4 min observation for the forced swim test). The cages were disinfected with an odorless disinfection solution after each mouse.

### 4.9. Data Analysis

The results are presented as means ± SD (standard deviation) or medians ± interquartile range (non-parametric analysis). The normality of data sets and their homogeneity were determined using D’Agostino and Pearson and Brown–Forsythe tests, respectively. The comparisons between experimental and control groups were performed by one-way ANOVA followed by Newman–Keuls *post hoc* or two-way ANOVA with repeated measures followed by Bonferroni *post hoc* test. One-sample t-test was used to analyze the results of the object recognition task. In cases when assumptions for normal distribution of data was not fulfilled, we used Kruskal–Wallis with Dunn’s *post hoc* test. A value of *p* < 0.05 was considered to be significant. 

## 5. Conclusions

In this study, we found that JJGW08, a novel arylpiperazine alkyl derivative of salicylamide, which is a strong antagonist of 5-HT_1A_ and D_2_ receptors and weak antagonist of 5-HT_2A_ and 5-HT_7_ receptors with the potential antipsychotic- and anxiolytic-like activity, possessed no affinity for 5-HT_6_ receptors, and no antidepressant-like activity in rodents. Furthermore, the compound did not impair mice’s long-term emotional or recognition memory acquisition and protected mice against the MK-801-induced recognition and emotional memory deficits. Therefore, our preliminary study may suggest that a blockade of serotonin receptors, especially 5-HT_1A_ and 5-HT_7_ receptors might be beneficial in treating cognitive impairments.

## Figures and Tables

**Table 1:** The influence of JJGW08 on locomotor activity in mice.

Treatment	Dose (mg/kg)	Number of Crossings ± SD	
		0–6 min	2–6 min
Saline	-	282 ± 91	196 ± 68
	0.15	164 ± 130	150 ± 61
	0.3	326 ± 85	214 ± 55
JJGW08	0.625	191 ± 81	131 ± 53
	1.25	155 ± 90	121 ± 50
	2.5	108 ** ± 136	70 ** ± 86

Locomotor activity was recorded separately for each mouse in actometers. After the 30-min adaptation period, the number of photobeams crossings was measured at the appropriate time intervals, i.e., 6 min for the tail suspension test and 4 min for the forced swim test. JJGW08 was administered intraperitoneally (*ip*) 30 min before the test. The control group received an injection of 0.9% NaCl (*ip*). Values are expressed as means ± SD, n = 8–10 mice per group. Statistical analysis: one-way ANOVA (Newman–Keuls *post hoc*), ** *p* < 0.01.

**Table 2:** Radioligand binding assay conditions.

Receptor	Radioligand/ Final Concentration	Blank (Non-Specific)	Buffer	Incubation Conditions
5-HT_6_	[^3^H]-LSD 2 nM	10 µM methiothepin	50 mM Tris–HCl pH 7.4 0.5 mM EDTA, 4 mM MgCl_2_	60 min, 37 °C

## Data Availability

Data are contained within the article.
